# Exploring the Experiences and Perceptions of Cigar Craving and Addiction among Young Adult Black Cigar Smokers

**DOI:** 10.3390/ijerph19116680

**Published:** 2022-05-30

**Authors:** Maryam Elhabashy, Lilianna Phan, Kristen R. Hamilton-Moseley, Aaron Broun, Danielle A. Duarte, Aniruddh Ajith, Bambi Jewett, Erin L. Mead-Morse, Kelvin Choi, Julia Chen-Sankey

**Affiliations:** 1 Center for Tobacco Studies, Rutgers Biomedical and Health Sciences, New Brunswick, NJ 08901, USA; maryam.elhabashy@rutgers.edu; 2 National Institute on Minority Health and Health Disparities, Division of Intramural Research, Bethesda, MD 20814, USA; lilianna.phan@nih.gov (L.P.); kristen.moseley@nih.gov (K.R.H.-M.); bambi.jewett@nih.gov (B.J.); kelvin.choi@nih.gov (K.C.); 3 Milken Institute School of Public Health, George Washington University, Washington, DC 20052, USA; abroun@gwmail.gwu.edu (A.B.); dduarte41@gwu.edu (D.A.D.); 4 School of Medicine, University of Pittsburgh, Pittsburgh, PA 15201, USA; ajith.aniruddh@medstudent.pitt.edu; 5 School of Medicine, University of Connecticut, Farmington, CT 06032, USA; mead@uchc.edu; 6 School of Public Health, Rutgers University, Piscataway, NJ 08854, USA

**Keywords:** cigar addiction, African Americans, health disparities, in-depth interviews, qualitative research

## Abstract

Black young adults have the highest prevalence of cigar smoking in the U.S. Little is known about how this population perceives addiction to cigar smoking, which may influence long-term smoking and cessation outcomes. This study used semi-structured in-depth interviews to understand cravings, triggers, and perceived addiction from cigar smoking among a purposive sample of Black young adult cigar smokers (*N =* 40; 21–29 years). An iterative process was used to develop the codebook, and thematic analysis was used to capture findings based on the products predominantly used: cigarillos, large cigars, or blunts. Results suggest that while participants may share similar types of cravings and triggers (e.g., stress) across the use of these products, predominant blunt smokers reported more unique triggers related to relieving physical discomforts. While most participants reported cigars could be addictive to people in general, only a few perceived that they themselves were addicted. Participants who predominantly smoked cigarillos reported high perceived addiction to cigars, while those who predominantly smoked blunts reported low addiction. Education messages are needed to inform young Black adult cigar smokers about the risks and health symptoms of cigar addiction. These efforts may help increase cigar cessation seeking and reduce cigar addiction-related health consequences and disparities among Black populations.

## 1. Introduction

Cigar smoking is associated with detrimental health outcomes, including oral, esophageal, pancreatic, laryngeal, and lung cancers [1]. However, health risks vary by cigar type (i.e., large cigars, cigarillos, filtered/little cigars, etc.), some of which can be more harmful to health than cigarettes [2]. Cigarillos, for example, can contain higher amounts of tobacco and more carcinogens per gram than cigarettes [3] and may promote nicotine dependence and expose users to a considerable amount of carbon monoxide [4]. Traditional large cigars may be more harmful than cigarettes, with the capacity to deliver 10 times the nicotine, two times the tar, and five times the carbon monoxide of cigarettes [2]. Although not all people who smoke cigars inhale while smoking, many of them do inhale just as with cigarette smoke, exposing themselves to substantial amounts of nicotine and harmful chemicals [1]. Even not inhaling cigar smoke may expose individuals who smoke cigars to health and addiction risks [1].

There is limited literature comparing the harm risks of respective cigar products relative to each other (there is much more research comparing cigar products to cigarettes). Nonetheless, there is research indicating that compared to filtered cigars, the composition of traditional large cigars contributes to increased oral absorption of carcinogenic constituents [5]. This may increase the risk of oral cavity cancers in large cigar users [6]. Of the various cigar products available to consumers, large cigars and cigarillos were also found to have the highest mean nicotine when compared to filtered cigars, pipe tobacco cigars, and mini cigarillos [7]. However, toxicants other than nicotine, such as acetaldehyde and benzene, are found to be more prevalent in cigarillo smoke compared to other cigar products [8]. Therefore, reducing cigar use could promote population health. 

Substantial racial disparities exist with tobacco use, particularly with cigar smoking. Black/African American individuals die from tobacco-related diseases at an 18% higher rate (~45,000 Black/African American lives) than White individuals in the U.S. annually [9], which in part could be attributed to cigar smoking. Between 2002 and 2016, U.S. Black adults had a significant increase in the prevalence of cigar smoking while the prevalence of cigar smoking remained stable among other racial/ethnic adult populations [10]. Black young adults have the highest prevalence of cigar smoking compared with any other racial/ethnic and age group in the U.S. [11,12,13]. Young adulthood is a critical developmental period in which tobacco experimentation occurs and can lead to establishment. Recent findings established that tobacco initiation has shifted from youth to young adulthood [14]. Additionally, between 2002 and 2018, the number of daily young adult cigarette smokers increased [15]. Therefore, interventions for preventing and reducing cigar smoking among young adults are greatly needed.

Additionally, nationally representative data from 2014 to 2015 showed that more than half of Black adults who currently smoked cigars had smoked blunts (defined as cigars that have been partially or wholly emptied of tobacco contents to be replaced with cannabis) in the past 12 months as compared to only about a quarter of White adults who currently smoked cigars [16]. Co-use and substitution of cigar products with cannabis are common among people who smoke cigarillos [17]. This co-use still exposes users to tobacco carcinogens; thus, blunt smoking may also be a risk factor for cancer [18]. An increasing number of states are legalizing cannabis for both medicinal and recreational use [19,20]. This may contribute to the growth of the cannabis market in the U.S., which may be evidenced by the increase in young adults’ rate of self-reported cannabis use since 2019 [21]. Cannabis use is also addictive, and concurrent use of tobacco and cannabis may further increase the symptoms of both cannabis and nicotine dependence [17,22,23]. 

Cigar smoking behavior is maintained by addiction, a condition that involves uncontrolled use of a substance despite its harmful consequences and is indicated by a cluster of cognitive, behavioral, and physiological symptoms [24]. Symptoms of cigar addiction, similar to cigarette addiction, may include cravings (i.e., intense urges to smoke that are often elicited by exposure to triggers), loss of control in limiting smoking, the development of tolerance (i.e., the need for increased amounts of the substance over time to achieve the desired effect), and withdrawal (i.e., the emergence of an aversive emotional and physiological state upon abrupt cessation of cigar smoking) [25,26]. Previous studies in the cigarette smoking literature suggest that a greater perceived risk of becoming addicted to cigarettes was associated with lower rates of continued smoking [27,28]. 

To date, however, little is known about how Black young adults perceive and experience cigar addiction. Moreover, interest in quitting appears to be low among Black young adult cigar smokers, highlighting the importance of understanding their experiences with addiction to inform interventions [29]. Investigating Black young adult cigar smokers’ perceived addiction to blunt smoking is also critical to understanding their overall experiences with cigar and cannabis addiction since these two products are often used together by cigar smokers [16]. Therefore, to address this knowledge gap and inform interventions and programs for reducing cigar smoking, this study used semi-structured in-depth interviews to explore perceptions of and experiences with addiction to cigar and blunt smoking among Black young adults who smoke cigars.

## 2. Materials and Methods

### 2.1. Recruitment: Participant Eligibility, Screening, and Consent

This study used in-depth telephone interviews with Black young adults who smoke cigars (*N =* 40) to understand their perceptions and experiences with cigar smoking and cigar addiction. For recruitment, the research team generated posts on social media platforms such as Facebook and Instagram. These posts targeted residents in Washington D.C. who met the following eligibility criteria: (1) self-identified as non-Hispanic Black or African American; (2) between the ages of 21 and 29 years old; (3) current cigar smokers (as defined by smoking premium cigars, cigarillos, or little cigars ≥ 4 times within the preceding 2 weeks) [16,29,30]; and (4) proficient in reading and speaking the English language.

All interested individuals were first screened by research team members by phone. Current cigar product smoking status was confirmed during the screening process by asking potential participants to describe the brands and characteristics of the cigar products they currently smoked. The research staff who screened the participants were trained to recognize cigar product brands and types. During the interview, participants were first asked to describe the brands and characteristics (including cigar product type and flavors) of the cigar products and cigar smoking patterns (e.g., frequencies) they currently smoked. Participants were also asked to describe their current blunt smoking behavior after the interviewer defined blunt products to them and assured them that the information would be kept confidential. 

Eligible individuals were subsequently emailed a link to an online survey, which contained informed consent and questions about sociodemographic characteristics (e.g., age, biological sex, educational attainment) and tobacco use history (e.g., past and/or current use of various tobacco products including cigars, cigarillos, cigarettes, and e-cigarettes). Participants who indicated they were willing to participate in the study and completed the survey questions were then contacted to schedule phone interviews. This study received an exempt determination from Institutional Review Board (IRB) review by the National Institutes of Health, Office of IRB Operations.

### 2.2. Data Collection: Participant Interviews

Phone interviews with participants (*N =* 40) were conducted by a research team member (JCS) with experience in conducting semi-structured in-depth interviews about tobacco use from May to June 2020. Interviews lasted between 45 and 60 min and took place over an audio-recorded phone call. Interviews were qualitative and in-depth in nature, as such characteristics make for honest and effective transmission and exchange of ideas pertaining to interviewee perspectives and experiences [31]. Participants received a USD 100 Amazon gift card after completing the phone interview. 

The interviewer asked the participants to discuss the following topics about cigar addiction: (1) experience in cigar smoking cravings and triggers; (2) perceptions that people, in general, could become addicted to cigar products; and (3) perceptions that they themselves are addicted to cigar products. These questions, which are presented in Table 1, were also tailored to the specific cigar products that participants reported smoking in the past 30 days (i.e., large cigars, cigarillos, filtered cigars, and blunts). The interviewer took notes during the interview in the form of key points and takeaways. Notes were used to evaluate data saturation and assess the point at which no new information was being generated from interviews [32]. While data saturation was reached by the 30th interview, additional interviews were conducted to confirm complete saturation.

### 2.3. Data Analysis

Pre-interview survey responses on socio-demographics and tobacco use histories were analyzed using Stata 16.0 (StataCorp LLC: College Station, TX, USA). Interview audio recordings were transcribed verbatim. Transcripts were then de-identified and analyzed using Dedoose, a web-based qualitative data management application (SocioCultural Research Consultants, LLC: Los Angeles, CA, USA). Next, four study team members (JCS, DD, AB, and AA) used an iterative process to develop codes based on topics of interest (i.e., cigar and blunt cravings, triggers, general perceptions of addiction, and self-perceived addictions) and emerging content from the interview data. Codes were further categorized for their relevance to four different cigar product types: large cigars, cigarillos, filtered cigars, and blunts. If the themes identified from the interviews had no notable differences across various cigar products, those themes were analyzed and presented with respect to overall cigar addiction or craving. If there were thematic differences across cigar products, they were analyzed and separated into product sub-sections of each theme. Three trained coders (DD, AB, and AA) applied the codebook to five same interview transcripts; they were invited to add codes to the codebook as needed, which were reviewed by the entire research team. A finalized codebook was agreed upon by the research team and applied to all 40 interview transcripts. Table 1 presents the codes used for this study and their definitions, as well as the interview questions associated with each.

Each transcript was independently coded by two of the three coders (DD, AB, and AA), who then met with a leading team member (JCS) to mediate any coding disagreements. The percentage agreement of the codes used for this analysis was moderate to high (84–92%), demonstrating satisfactory inter-coder reliability [33]. Reporting of themes and subthemes was based on the frequencies of relevant content discussed by the participants using the following words: “all” (100%), “most” (70–99%), “more than half” (51–69%), “half” (50%), “some” (20–49%), “a few” (1–19%), and “none” (0%) [16,29,34,35]. Due to the limited amount of interview data specific to filtered cigar-smoking, themes specific to filtered cigars were not generated or reported. Exemplary quotes were provided while noting the sexes, ages, and most frequently used (predominant) cigar products of the participants.

## 3. Results

### 3.1. Participant Characteristics from the Pre-Interview Survey

The mean age of participants was 26 years (SD = 1.5 years). Females made up slightly more than half of the sample (*n =* 23, 57.5%) (Table 2). During the 30 days prior to the pre-interview survey, participants reported smoking cigarillos (*n =* 36, 90%), large cigars (*n* = 24, 60%), blunts (*n =* 23, 57.5%), and filtered cigars (*n =* 7, 17.5%). Regarding the most frequently used cigar product in the past 30 days, almost half of the participants reported blunts as their predominant cigar product (*n =* 18, 45%), followed by cigarillos (*n =* 16, 40%), large cigars (*n =* 4, 10%), and filtered cigars (*n =* 2, 5%). More than half of the participants also reported using other tobacco products in the past 30 days (*n =* 27, 67.5%). Additionally, past 30-day use of cigarettes, e-cigarettes, and hookah was reported by 23 (57.5%), 26 (65.0%), and 27 (67.5%) participants, respectively.

### 3.2. Cigar Smoking Cravings

Most participants described that they experienced cravings for cigar products in general. Specifically, some participants described cravings for nicotine satiation in general, while others described specifically feeling a craving for the physical habits and sensations associated with cigar smoking behavior.

*“No, I don’t see a difference [in cravings across cigar products]. If I want to smoke, I’ll smoke anything that I have. Anything.”*

(Male, 28 years, large cigars)

*“Honestly, the action of smoking, the repetition of it, just having a J [blunt] in my hand and having the feeling of the inhale and the exhale of the smoke, is something that’s also appealing to me while I smoke.”*

(Female, 28 years, blunts)

*“I do have the urge. I don’t think it’s the urge to smoke Black & Milds. It’s just nicotine. I can stop smoking the Milds [Black & Milds] but I’ll still smoke a vape or I’ll smoke a hookah or something that has nicotine in it. It’s not just Blacks [Black & Milds] that I’m loyal to. It’s really the nicotine, is what it is.”*

(Male, 27 years, cigarillos)

A few predominant blunt smokers also reported that cravings for smoking blunts for cannabis were generally stronger than cravings for nicotine or that it was difficult to differentiate between cravings for cannabis or nicotine.

*“It’s more a craving of weed because you don’t want to crave too much tobacco. It has chemicals and it’s not too good for you. The weed is the real craving. It has psychoactive effects and stuff, so that’s the real craving.”*

(Male, 28 years, blunts)

*“I think sometimes it’s hard to tell [what I feel the stronger urge for] because I’m putting the marijuana into the tobacco.”*

(Female, 25 years, blunts)

### 3.3. Cigar Smoking Triggers

When asked about cigar smoking triggers, most participants mentioned contextual (e.g., spending time with friends), psychological (e.g., stress), and behavioral (e.g., waking up) triggers. Some triggers experienced by the participants were consistent across various cigar products: time of day, anxiety, depression, stress, boredom, socializing, smelling cigar smoke or blunt smoke in the air, and seeing someone smoking cigars.

*“Yes, I really think the end of the day may be a trigger. I was going to say earlier, it’s a mind thing. I’m used to smoking after work so when I get off work, maybe it’s not even the day but I’m just used to smoking at that time.”*

(Male, 27 years, cigarillos)

*“I struggle with anxiety, so if I start to feel anxious or stressed or overwhelmed about something, I would turn to smoking. If I had a very crazy day and my day was overwhelming and stressful, then I would turn into it. I guess mostly just being under stress triggers me to smoke, or just being anxious.”*

(Female, 25 years, blunts)

*“I guess there’s a certain element of just wanting to share in the same activity as your friends as well that makes you want to do it.”*

(Female, 25 years, blunts)

*“It’s a trigger because if I see somebody have one [cigarillo], I’m like, ‘I want to have one…’ It’s a trigger. I don’t know why.”*

(Female, 28 years, cigarillos)

Some triggers discussed by the participants, especially those for predominant cigarillo smokers and blunt smokers, are product-specific, including pairing certain cigarillo flavors with food or meals. Being in an environment where smoking blunts could lead to social and/or legal consequences was also described as a trigger for non-blunt cigar smoking.

*“I do the vanilla [flavor] in the morning… It reminds me of having a coffee with vanilla in it in the morning… Then the wine [flavor] I’ll do after a meal. For some reason it reminds me of having a glass of wine or something like that.”*

(Female, 29 years, cigarillos)

*“It just makes you feel a little bit more satisfied after you eat it because it’s a big meal. That’s the main thing.”*

(Male, 27 years, cigarillos)

*“I’ve tried to stop and it’s hard when you always have access [to cigarillos]. Yes, that’s definitely one of them [triggers]… The tobacco in the cigars, you can smoke them everywhere. You can’t smoke a blunt everywhere.”*

(Female, 27 years, blunts)

Regardless of the predominant cigar product smoked, more product-specific triggers were identified for blunts than for any other product in this study. Some participants described them as triggers for consuming cannabis rather than tobacco or nicotine. Product-specific triggers for predominant blunt smokers included feeling pain, a lack of energy, a lack of creative inspiration, and low morale. A few participants described the medicinal qualities of cannabis as a significant trigger for smoking blunts. Some reported the desire to “get high” as a significant trigger for them to smoke.

*“I use marijuana for medical purposes. I have endometriosis and fibroids. I use the marijuana to calm those so I’m not taking Tylenol, Naproxen, and Advil all day, every day.”*

(Female, 29 years, blunts)

*“Blunts, like I said is an everyday thing. I like the high of a blunt so that’s something I do every day. The trigger for a blunt is to X the day out.”*

(Female, 29 years, blunts)

*“If I’m frustrated with creation by creating something like a project or painting something or making music, I’ll smoke a blunt.”*

(Male, 27 years, cigarillos)

### 3.4. General Perceptions of the Addictive Potential of Cigars

When asked whether people can become addicted to cigars in general, most of the participants believed that people could become addicted to cigar products. These beliefs were almost unanimous across smokers of all cigar products. Some of these participants expressed that cigar products were addictive since people can become addicted to any tobacco product by virtue of its nicotine content. A few stated that cigar products could be addictive since smoking them became part of people’s routines.

*“Nicotine is an ingredient in both [cigarillos and blunts] and nicotine is an addictive chemical… Anything as far as tobacco, there is a risk of being addicted because of the nicotine and the effects that nicotine has on the brain.”*

(Female, 29 years, blunts)

*“Because it [cigar product] has tobacco in it and tobacco is one of the most addictive drugs in the world, ma’am.”*

(Female, 25 years, cigarillos)

*“People tend to get addicted to any type of smoking… Once they’ve started and I feel like it’s a routine that they want to do. For some reason, you just don’t want to stop.”*

(Male, 28 years, large cigars)

A few participants did not believe that people could become addicted to cigar products. Specifically, they stated that they did not know of anyone who was addicted to cigars since they were only smoked for recreational purposes. A few also perceived that there was either no nicotine or not enough nicotine in cigar products to lead to cigar addiction. A few stated that self-control ensured that people could not become addicted to cigar products.

*“I personally don’t know anybody that’s truly addicted to cigars. I know people that smoke on a daily basis because they enjoy it and they like the environment, and they may feel like it’s just a relaxing experience and they need that relaxation, but I don’t think that I know anyone that’s truly addicted to it, where their body needs it.”*

(Female, 26 years, large cigars)

*“I don’t think [people can become addicted to cigar products] because I don’t think there’s any nicotine in it.”*

(Male, 27 years, cigarillos)

*“Self-control. Addiction is about control. If you don’t have control, you don’t need to be doing it. That’s how I feel. I could stop right now if I wanted to. Even if everybody is smoking, if I really, really wanted to stop I would stop.”*

(Female, 27 years, blunts)

### 3.5. Self-Perceptions about Cigar Addiction

When participants were asked whether they believed themselves to be addicted to the cigar products they used, more than half stated that they were not addicted because they could “stop smoking” anytime or that they felt no “physical addiction” in the form of symptoms. These beliefs were especially prevalent among predominant blunt smokers, most of whom identified themselves as not addicted to cigar products. Some blunt smokers maintained that they had full self-control over their bodies and thus were not addicted. A few of them asserted that cannabis has psychological effects that do not develop into an addiction like nicotine.

*“I don’t feel like my body reacts to it [blunts] like, “Oh, you need to smoke…” You know how people have a nicotine addiction? It’s nothing like that…It’s more so a mental thing.”*

(Female, 23 years, blunts)

*“I’m fine. You can control your own body. I highly believe in that. It’s your body and you control your own body. I would be just fine without it [blunts] too.”*

(Male, 29 years, blunts)

*“As of now, I don’t feel like I can’t function without weed, or I can’t go a day without smoking weed, or anything like that. I can go a whole week without smoking weed if I want to, or more than that. I don’t think there’s an addiction there.”*

(Female, 23 years, blunts)

In contrast, most predominant cigarillo smokers identified themselves as addicted to cigar products. Some of these individuals reported that they believed themselves to be addicted because they felt a loss of self-control, observed that smoking cigarillos became part of their daily routine, or felt that the nicotine content of cigarillos made them addicted.

*“It [smoking cigarillos] seems like something I need every day in my life. It’s like if somebody needs a cup of coffee. You know how people have to have their coffee every day? It’s almost like a caffeine addiction, but not caffeine.”*

(Female, 28 years, cigarillos)

*“Because I think that I started off with one and now I’ve moved up to two, three a day. It’s just a part of your routine. Even though I don’t feel like a drug addict where if I go a day without I’m going to be panicky. It is a part of a daily routine and it’s hard to break daily routines. It’s pretty hard when you are doing it consistently, everyday basis.”*

(Female, 29 years, cigarillos)

*“It’s [nicotine] a neurotransmitter and when you have the cigarette it stimulates that part of your brain… That’s kind of another reason that if I’m having a stressful problem that I’m smoking these things [cigarillos] back-to-back.”*

(Male, 28 years, cigarillos)

## 4. Discussion

This is one of the first studies to explore cigar smoking-related cravings, triggers, and addiction among Black young adults who smoke cigars. Results from this study suggest that while Black young adults who smoke cigars may share similar beliefs about cravings, triggers, and general perceptions about cigar addiction, their self-perceptions about cigar addiction may vary significantly depending on whether they use cigar products to consume tobacco (i.e., cigarillos, large cigars) or cannabis (i.e., blunts). Notably, most participants reported experiencing symptoms that are consistent with those from cigar addiction (e.g., cravings and withdrawal symptoms), regardless of the recognition of their own cigar addiction. Most participants reported cigar-related triggers, with people who predominantly smoked blunts reporting more unique triggers than users of other cigar products, such as the desire to relieve physical discomforts from using cannabis. Additionally, although almost all participants believed people, in general, could become addicted to cigar products, almost half did not identify themselves as experiencing addiction to cigar smoking. Specifically, people who predominantly smoked blunts were least likely to identify themselves as addicted to cigars, while people who predominantly smoked cigarillos were most likely to identify themselves as addicted to smoking cigars.

Many of the cigar smoking-related triggers identified by participants were aversive psychological states such as distress, anxiety, and depression. This is consistent with previous research that found affect as a significant trigger for cigar smoking among Black cigar smokers [30,35]. Notably, our study was conducted during the COVID-19 pandemic, during which aversive psychological states may be heightened. This study is one of the first to identify a similar relationship among cigar smokers who often described aversive psychological states as triggers for smoking to ease their symptoms. This coping mechanism is especially concerning, given that some cigar products can contain a higher amount of nicotine than cigarettes [8]. Previous studies found that prolonged nicotine use and dependence can worsen mental health symptoms, including anxiety and depression [36], and these symptoms may further trigger the increased use of nicotine and tobacco products [37,38]. In addition to a few participants in our study misperceiving that there was little to no nicotine in cigars, these findings indicate the crucial need for effective messaging communicating the addiction risks of cigar smoking to Black young adults. 

Among participants who predominantly smoked blunts, relieving physical discomforts (e.g., pain) or seeking desirable medical outcomes were described as triggers for smoking cigars. This belief, coupled with a lack of understanding that cannabis is addictive and blunts also contain nicotine, may lead Black young adults to continue smoking blunts. Past research has suggested that cannabis use is associated with cigar addiction in Black cigar smokers [39]. Thus, as blunt smoking becomes an increasingly common way to consume cannabis, especially among Black young adults [40], it is imperative to inform people who predominantly smoke blunts about the harm and risk of developing not only cannabis use dependence and disorders but also nicotine addiction and its associated health consequences. 

In our study, while most participants believed that cigar products could be addictive to people in general, much fewer participants identified themselves as being addicted to cigars. This observed dissonance in cigar addiction perceptions may lower cigar smokers’ perceived severity of engaging in cigar smoking behavior, which may eventually reduce their likelihood of quitting cigar products. Additionally, although most participants reported cigar smoking cravings, which is a notable symptom of cigar addiction, only some reported self-addiction. This discrepancy may be largely due to participants’ lack of understanding of nicotine and tobacco addiction symptoms. Furthermore, our finding that more than half of predominant cigarillo smokers identified themselves as addicted to cigar products is consistent with a recent study exploring self-perceptions of addiction among adolescent and young adult cigarillo smokers [41]. Black young adults who predominantly smoked cigarillos were also more likely to report interests and motivations in quitting cigar products than smokers of other cigar and blunt products [29]. Therefore, interventions for treating cigar addiction and cessation can first target cigarillo smokers who may already be aware of their addiction to the products and are interested in quitting smoking. 

We found that perceived addiction to cigar products may be the lowest among blunt smokers, which builds on previous research that found that blunt smokers have low perceptions of harm from cigar smoking and little interest in quitting smoking [29]. Together, our findings indicate a lack of knowledge among our participants that smoking blunts may result in harm and have risks associated with smoking combustible tobacco products as well as cannabis [18,42]. Thus, public education messaging is needed to communicate that blunt smoking can contribute to nicotine, cannabis, and tobacco addiction and harm. More research on the rationale behind these findings is needed to inform strategies for reducing blunt smoking among Black young adults. The information from this in-depth interview study can inform large-scale, quantitative surveys that may serve to predict and measure such concepts as cigar addiction and craving among this priority population.

### Study Limitations

This study has the following limitations. First, data collection took place during the beginning of the COVID-19 pandemic. Such strenuous circumstances could have increased cigar use and perceived cigar addiction due to escalated mental stress [16]. Second, participants were mostly from the Washington D.C. area, where recreational cannabis has been decriminalized [43]. Therefore, findings might not be representative of Black young adult cigar smokers living in localities where cannabis policies and legalities vary. The qualitative nature of the study and convenience sampling further limit its generalizability. Third, this study did not specifically explore various types of addiction symptoms among participants. Although some participants mentioned their experiences with cigar smoking withdrawal and loss of control, none discussed building cigar-smoking tolerance over time. Additionally, only one team member was tasked with conducting the interviews and thus may have asked questions differently than other potential interviewers. This could potentially bias results or limit the scope of questions asked. Finally, this study did not specifically ask participants to discuss their perceived cannabis dependence or addiction, although some participants brought up their cravings for cannabis themselves. Future studies should examine perceived addictiveness of and addiction to both nicotine and cannabis products among cigar smokers to better inform cigar smoking-related interventions that may need to address cannabis dependence.

## 5. Conclusions

Considering the heightened prevalence of cigar smoking among Black young adults—a group grappling with numerous tobacco-related health disparities—the promotion of cigar cessation and cigar addiction treatment is of particular importance. The results from this study shed light on the importance of acknowledging varying perceptions of addictiveness and addiction towards different cigar and blunt products. Increasing knowledge and risk perceptions about cigar addiction among Black young adult cigar smokers may be an effective step toward reducing cigar smoking and its health outcomes among Black communities. Finally, those interventions and programs may also need to especially consider addressing cannabis addiction, cravings, and triggers among cigar and blunt smokers.

## Figures and Tables

**Table 1:** Codebook Used for Identifying and Analyzing Emergent Themes in Interviews.

Interview Question (s)	Code ^1^	Definition
Do you ever have strong cravings or urge to smoke cigars? Could you describe what it feels like?	Craving	Urges or desires to engage in smoking behavior, independent of triggers
What usually makes you want to smoke cigars? What are your triggers?	Trigger	Cues and stimuli that instigate smoking episodes
Would you say that people in general can become addicted to smoking cigars? And why?	Addiction	General perceptions of whether participants believe cigar products can be addictive
Do you think you are addicted to smoking cigars? And why?	Addicted	Individual perceptions of whether participants identify themselves as addicted to cigar products

^1^ Within each code category, subcodes were generated for each cigar product type predominantly smoked by participants.

**Table 2:** Participant Characteristics (*N =* 40).

	n	%
Age (mean, SD ^1^)	26.0	2.4
Biological Sex		
Male	17	42.5%
Female	23	57.5%
Education Level		
≤GED ^2^ or high school	7	17.5%
Some or completed technical school	9	22.5%
Some college	15	37.5%
≥Bachelor’s degree	9	22.5%
Current Employment Status		
Full time	19	47.5%
Part time	7	17.5%
Unemployed	11	27.5%
Others	3	7.5%
Current Financial Situation		
Live comfortably	13	32.5%
Meets needs with a little left	15	37.5%
Just meet basic expenses	12	30.0%
Cigar Smoking in the Past 30 Days		
Blunts	23	57.5%
Cigarillos	36	90.0%
Large cigars	24	60.0%
Filtered cigars	7	17.5%
Number of Cigar Products Smoked in the Past 30 Days		
One product	4	10.0%
Two products	16	40.0%
Three products	11	27.5%
Four products	9	22.5%
Most Frequently Smoked Cigar Product in the Past 30 Days		
Blunts	18	45.0%
Cigarillos	16	40.0%
Large cigars	4	10.0%
Filtered cigars	2	5.0%
Use of Other Tobacco Products in the Past 30 Days		
Cigarettes	23	57.5%
E-cigarettes	26	65.0%
Hookah	27	67.5%

^1^ SD: Standard Deviation. ^2^ GED: General Educational Development (a high school equivalency diploma in the U.S.).

## Data Availability

Interview transcripts and other relevant data are available upon request.
